# Dialysis symptom index burden and symptom clusters in a prospective cohort of dialysis patients

**DOI:** 10.1007/s40620-022-01313-0

**Published:** 2022-04-16

**Authors:** Amy S. You, Sara S. Kalantar, Keith C. Norris, Rene Amel Peralta, Yoko Narasaki, Ronald Fischman, Michael Fischman, Avedik Semerjian, Tracy Nakata, Zahra Azadbadi, Danh V. Nguyen, Kamyar Kalantar-Zadeh, Connie M. Rhee

**Affiliations:** 1 Division of Nephrology, Hypertension, and Kidney Transplantation, University of California Irvine School of Medicine, 101 The City Drive South, City Tower, Orange, CA 92868 USA; 2 University of California Berkeley, Berkeley, CA USA; 3 Department of Medicine, David Geffen School of Medicine at UCLA, Los Angeles, CA USA; 4 Southland Renal Group, Lakewood, CA USA; 5 University of California Irvine School of Pharmacy and Pharmaceutical Sciences, Irvine, CA USA; 6 Division of General Internal Medicine, University of California Irvine School of Medicine, Orange, CA USA

**Keywords:** Unpleasant symptoms, Symptom clusters, Uremia, Dialysis

## Abstract

**Background:**

Dialysis patients experience a high symptom burden, which may adversely impact their quality of life. Whereas other specialties emphasize routine symptom assessment, symptom burden is not well-characterized in dialysis patients. We sought to examine the prevalence and severity of unpleasant symptoms in a prospective hemodialysis cohort.

**Methods:**

Among 122 hemodialysis patients from the prospective *Malnutrition, Diet, and Racial Disparities in Chronic Kidney Disease (CKD)* study, CKD-associated symptoms were ascertained by the Dialysis Symptom Index, a validated survey assessing symptom burden/severity (with higher scores indicating greater symptom severity), over 6/2020–10/2020. We examined the presence of (1) individual symptoms and symptom severity scores, and (2) symptom clusters (defined as ≥ 2 related concurrent symptoms), as well as correlations with clinical characteristics.

**Results:**

Symptom severity scores were higher among non-Hispanic White and Hispanic patients, whereas scores were lower in Black and Asian/Pacific Islander patients. In the overall cohort, the most common individual symptoms included feeling tired/lack of energy (71.3%), dry skin (61.5%), trouble falling asleep (44.3%), muscle cramps (42.6%), and itching (42.6%), with similar patterns observed across racial/ethnic groups. The most prevalent symptom clusters included feeling tired/lack of energy + trouble falling asleep (37.7%); trouble falling asleep + trouble staying asleep (34.4%); and feeling tired/lack of energy + trouble staying asleep (32.0%). Lower hemoglobin, iron stores, and dialysis adequacy correlated with higher individual and overall symptom severity scores.

**Conclusion:**

We observed a high prevalence of unpleasant symptoms and symptom clusters in a diverse hemodialysis cohort. Further studies are needed to identify targeted therapies that ameliorate symptom burden in CKD.

**Graphical abstract:**

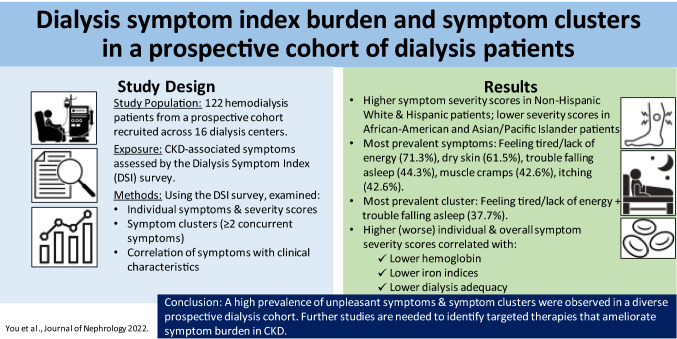

**Supplementary Information:**

The online version contains supplementary material available at 10.1007/s40620-022-01313-0.

## Introduction

Patients with kidney failure, including those who receive maintenance dialysis therapy, experience a high prevalence of physical and emotional disease-related symptoms [1–5]. A high symptom burden has been associated with higher death risk in dialysis patients [6], as well as lower health-related quality of life (HRQOL) vis-à-vis interference with their activities of daily living and physical function, independence, social relationships and activities, and overall well-being [7, 8]. Moreover, dialysis patients oftentimes prioritize alleviation of symptoms over other health outcomes such as survival and biochemical indices [8].

Despite the high burden of unpleasant symptoms in end-stage renal disease (ESRD), they are frequently under-recognized, under-estimated, and consequently under-treated [2, 9]. Ascertainment of chronic kidney disease (CKD)-associated symptoms in dialysis patients may be challenging due to their vague nature, difficulty in objective quantification, and conflation with other co-existing comorbidities [2]. Additionally, limited patient—provider interaction time and reporting biases influenced by social/cultural factors may prelude adequate symptom assessment, leading to some patients potentially adapting to and/or minimizing their symptoms over time [10].

The use of validated assessment tools may facilitate communication between patients and their providers about the presence and/or severity of their symptoms [5]. There are multiple generic and disease-specific instruments for symptom evaluation, among which the Dialysis Symptom Index, a comprehensive 30-item survey assessing the presence and severity of unpleasant symptoms [11, 12], is the most frequently used instrument utilized in CKD and ESRD patients. While an increasing number of epidemiologic studies have sought to use the Dialysis Symptom Index to uncover the prevalence of physical and emotional symptoms in US dialysis patients [6, 11–19], comparisons of symptom burden across diverse racial/ethnic groups remain incompletely characterized.

Furthermore, whereas emerging data in other fields (i.e., oncology) show that unpleasant symptoms often occur in clusters (i.e., defined as two or more concurrent symptoms that are related to one another, which may have a more significant effect than isolated symptoms) [1, 20], there has been limited study of symptom clusters in ESRD patients receiving dialysis. To address this knowledge gap, we sought to examine the prevalence of individual symptoms, as well as pairings of symptoms within specific symptom clusters in a racially/ethnically diverse, multi-center prospective cohort from the NIH *Malnutrition, Diet and Racial Disparities in Chronic Kidney Disease (MADRAD)* study [21–24] who underwent protocolized Dialysis Symptom Index survey assessments.

## Methods and materials

### Study population

The study population was a cohort of adult hemodialysis patients from the *MADRAD* study [21–24] (ClinicalTrials.gov NCT01415570), an ongoing prospective cohort study in which the impact of dietary and nutritional intake patterns upon outcomes across different racial/ethnic groups is being examined. In the MADRAD study, participants undergo protocolized collection of information on socio-demographic backgrounds, comorbidities, dialysis treatment characteristics, and validated surveys of patient-centered outcomes (e.g., Dialysis Symptom Index) every six months (i.e., every semester).

In this cross-sectional substudy, we assessed dialysis patients’ (1) individual symptom burden, ascertained by Dialysis Symptom Index surveys, as well as (2) symptom clusters among hemodialysis patients recruited across 16 outpatient dialysis facilities in Southern California over the period of June to October 2020. Patients were included provided that they completed at least one or more Dialysis Symptom Index surveys, were at least 18 years of age at the time of study entry (i.e., time of baseline Dialysis Symptom Index assessment), and had been receiving in-center hemodialysis for at least four consecutive weeks. Patients were excluded if they were actively receiving peritoneal dialysis, had a life expectancy of less than six months (i.e., defined as having a terminal disease such as metastatic cancer, or enrollment in hospice while concurrently receiving dialysis), or were unable to provide consent without a proxy. The study was approved by the Institutional Review Board committee at the University of California Irvine.

### Dialysis symptom index assessment

In this study, we examined baseline Dialysis Symptom Index surveys, which were collected from in-center hemodialysis patients during their routine hemodialysis treatments. The Dialysis Symptom Index is a validated instrument to assess CKD-related unpleasant symptoms, and it is comprised of 30 questions about the presence of specific symptoms [11, 12]. For each question querying presence vs. absence of a specific symptom, symptom severity is assessed using a five-point Likert scale, with each response ranging from 0 to 4 (i.e., a response of “0” indicates “no,” whereas a response of “4” indicates “yes: very much”). The minimum–maximum Dialysis Symptom Index score ranges from 0 to 120, with higher scores indicating greater overall severity of symptoms.

In primary analyses, we examined (1) the clinical characteristics of patients reporting higher vs. lower symptom burden defined by overall symptom severity scores, as well as (2) the prevalence of individual symptoms from the Dialysis Symptom Index surveys. In secondary analyses examining groupings of symptoms, we also assessed (3) the prevalence of pairings of symptoms within specific symptom clusters categorized according to end-organ domains, namely the gastrointestinal, dermatologic, psychiatric, sleep, pain, neurologic, and endocrine systems, as well as (4) correlations of individual symptoms from the Dialysis Symptom Index surveys.

### Socio-demographics, comorbidities, and dialysis treatment characteristics

At study entry, baseline information on socio-demographics, comorbid conditions, and dialysis treatment characteristics (e.g., vascular access type) were collected. Dialysis vintage was defined as the time period between the date of study entry (i.e., date of baseline Dialysis Symptom Index assessment) and the date of hemodialysis initiation. Routine dialysis laboratory measurements were performed by the outpatient dialysis laboratories on a monthly or quarterly basis using automated methods. Information on race/ethnicity was self-reported by participants.

### Statistical analyses

Baseline characteristics between exposure groups (e.g., higher/lower symptom burden) were compared using chi-square, analysis of variance, and Kruskal–Wallis tests according to variable type. We first examined the distribution of the Dialysis Symptom Index severity scores and prevalence of individual symptoms in the overall cohort. In order to determine if symptoms differed across race/ethnicity, we also separately examined score distributions and prevalence of individual symptoms across racial/ethnic groups. We then assessed the prevalence of pairings of symptoms within specific symptom clusters categorized according to groupings by end-organ system. We additionally examined correlations of individual symptoms from the Dialysis Symptom Index surveys using Pearson and Spearman correlations adjusted for age, sex, and race/ethnicity. In sensitivity analyses of a subcohort of patients who had measurement of key laboratory parameters (i.e., hemoglobin, iron, dialysis adequacy, nutritional, mineral bone disease, and electrolyte values) within six months prior to or within one month after the Dialysis Symptom Index survey assessment, we examined correlations between the most proximate clinical laboratory value with overall symptom severity and individual symptom severity scores. The analyses and figures were generated using SAS version 9.4 (SAS Institute Inc., Cary, NC, USA), Stata version 13.1 (Stata Corporation, College Station, TX, USA), and SigmaPlot version 12.5 (Systat Software, San Jose, CA, USA).

## Results

### Baseline characteristics

After applying the eligibility criteria, the final study cohort was comprised of 122 hemodialysis patients, with a mean ± SD age of 60 ± 13 years, among whom 8% were non-Hispanic White, 62% were Hispanic White, 22% were Black, 6% were Asian/Pacific Islander, and 2% were of Other race/ethnicity (Table 1). In the overall cohort, the median (IQR) and minimum–maximum Dialysis Symptom Index overall symptom severity scores were 24 (11, 36) and 0–100, respectively (Supplementary Table 1). Among the thirty symptoms queried in the Dialysis Symptom Index, the median (IQR) and minimum–maximum number of individual symptoms reported by patients was 9 (5, 13) and 0–30, respectively.

**Table 1:** Baseline characteristics according to tertiles of baseline Dialysis Symptom Index (DSI) scores

	Overall	Tertile 1	Tertile 2	Tertile 3	P-value
N (%)	122	40 (33)	39 (32)	43 (35)	N/A
DSI score (min–max)	0–100	< 15	15- < 32	≥ 32	N/A
Age (mean ± SD), years	60 ± 13	61 ± 12	58 ± 16	60 ± 12	0.50
Female (%)	51	43	54	56	0.43
Race/ethnicity (%)					
Non-Hispanic White	8	5	8	12	0.72
Hispanic White	62	58	62	67	
Black	22	30	23	14	
Asian/Pacific Islander	6	5	8	5	
Other	2	3	0	2	
Diabetes (%)	67	70	59	72	0.41
Vintage (mean ± SD), months	81 ± 80	81 ± 50	69 ± 48	90 ± 117	0.50
Access (%)					
AVG/AVF	93	95	90	95	0.59
Catheter	7	5	10	5	
Marital status (%)					
Married	37	45	31	35	0.20
Single	37	43	36	33	
Other	26	13	33	33	
Insurance (%)					
Medicare/Medicaid	84	83	82	86	0.48
Kaiser, Private	11	8	15	12	
Other	5	10	3	2	
BMI (mean ± SD), kg/m^2^	30.1 ± 7.1	31.4 ± 7.3	28.7 ± 7.8	30.2 ± 5.9	0.37
Post weight (mean ± SD), lbs	181 ± 49	191 ± 44	171 ± 57	182 ± 44	0.34

SD, standard deviation; AVG/AVF, arteriovenous graft/arteriovenous fistula; BMI, body mass index; kg/m^2^, kilogram/meter squared; lbs, pounds

Baseline characteristics of the cohort stratified by baseline Dialysis Symptom Index overall symptom severity scores are shown in Table 1. Compared to patients in the lowest (first) Dialysis Symptom Index severity score tertile, those in the highest (third) tertile were more likely to be female, non-Hispanic White, and Hispanic White; were less likely to be Black; had longer dialysis vintages; were less likely to be married or single; and were more likely to have Medicare/Medicaid as their primary insurance.

Upon comparing the distribution of Dialysis Symptom Index symptom severity scores across racial/ethnic groups, median (IQR) values were higher in non-Hispanic White and Hispanic White patients (26 [16, 54] and 25 [12, 37], respectively), yet tended to be lower in Black and Asian/Pacific Islander patients (22 [9, 30]) and 19 [14, 46], respectively) as shown in Supplementary Table 1. Additionally, the median (IQR) number of individual symptoms reported by patients tended to be higher in non-Hispanic White and Hispanic White patients (11 [5, 19] and 10 [6, 14], respectively), yet were lower in Black and Asian/Pacific Islander patients (7 [4, 10] and 9 [6, 13], respectively). Supplementary Table 2 shows the baseline characteristics of the cohort stratified by racial/ethnic group.

### Individual symptoms in the overall cohort and across race/ethnicity

**Fig. 1:**
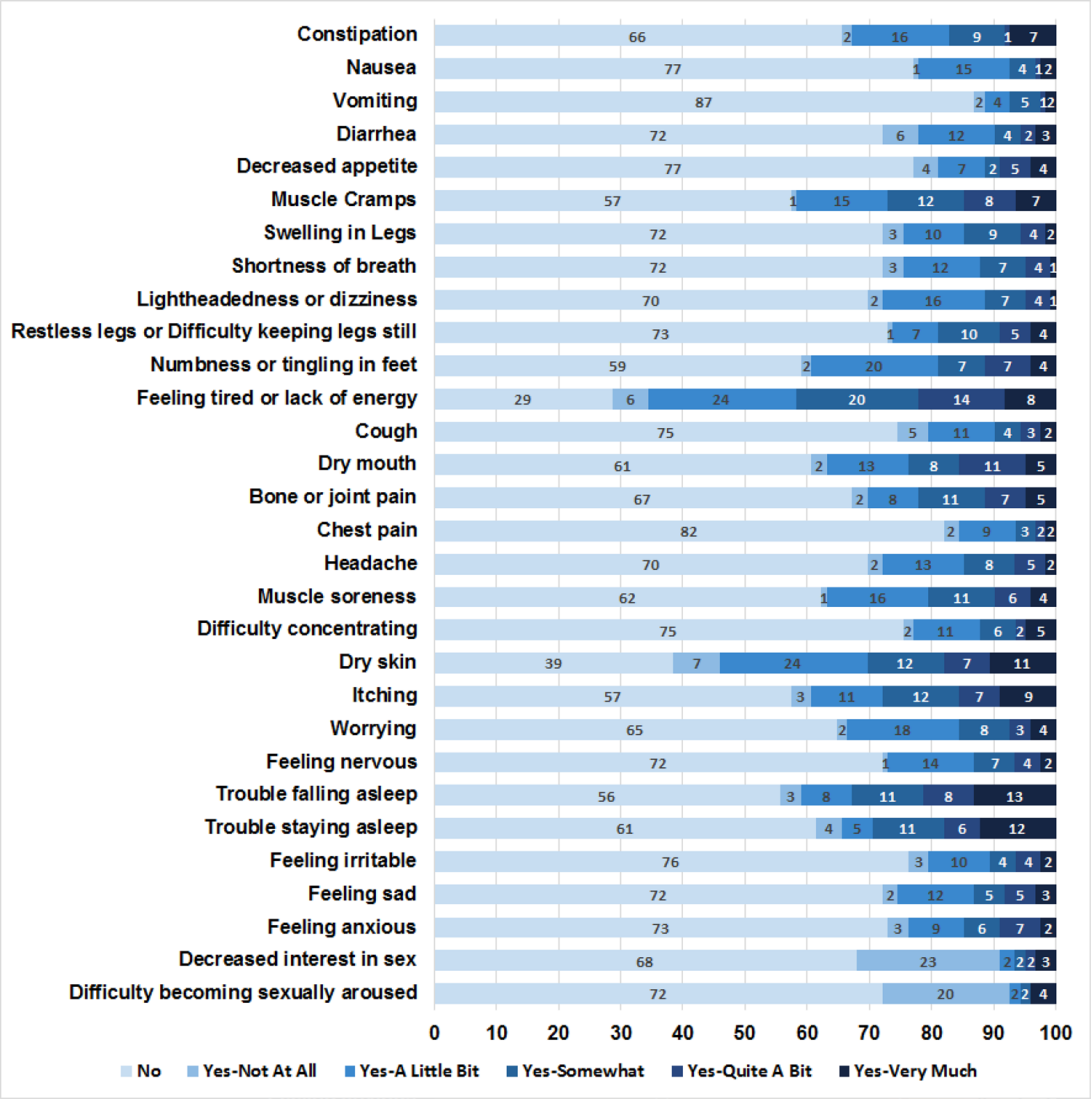
Prevalence of individual symptoms in the overall cohort

**Fig. 2:**
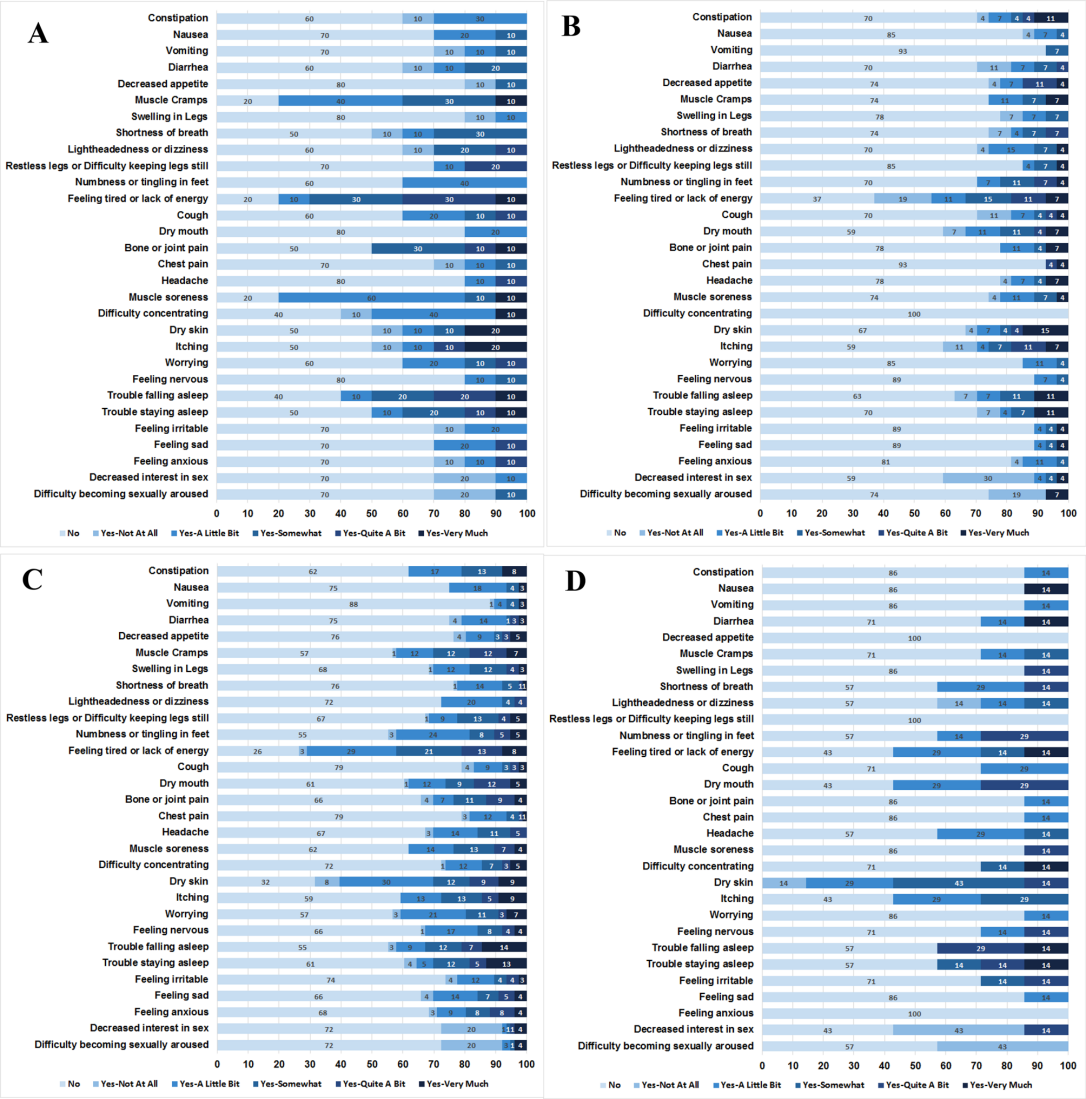
Prevalence of individual symptoms across racial/ethnic groups: Non-Hispanic White (**A**), Black (**B**), Hispanic White (**C**), and Asian/Pacific Islander (**D**)

The prevalence of individual symptoms from the Dialysis Symptom Index were examined in the overall cohort, among whom the most common symptoms reported included feeling tired or lack of energy (71.3%), dry skin (61.5%), trouble falling asleep (44.3%), muscle cramps (42.6%), and itching (42.6%) (Fig. 1). Given the abovementioned differences in the distribution of Dialysis Symptom Index scores across racial/ethnic groups, we also compared the burden of individual symptoms among patients of non-Hispanic White, Hispanic White, Black, and Asian/Pacific Islander background (Fig. 2A–D). Among non-Hispanic White hemodialysis patients, the most prevalent symptoms included muscle cramps (80.0%), muscle soreness (80.0%), feeling tired or lack of energy (80.0%), trouble falling asleep (60.0%), and difficulty concentrating (60.0%). However, among Black hemodialysis patients, the most common symptoms included feeling tired or lack of energy (63.0%), dry mouth (40.7%), itching (40.7%), decreased interest in sex (40.7%), and trouble falling asleep (37.0%). Yet in Hispanic hemodialysis patients, the most frequent symptoms were feeling tired or lack of energy (73.7%), dry skin (68.4%), trouble falling asleep (43.4%), numbness or tingling in feet (44.7%), muscle cramps (43.4%), or worrying (43.4%). Among Asian/Pacific Islander hemodialysis patients, prevalent symptoms included dry skin (100%), feeling tired or lack of energy (57.1%), dry mouth (57.1%), itching (57.1%), and decreased interest in sex (57.1%).

### Symptom clusters and correlations

**Fig. 3:**
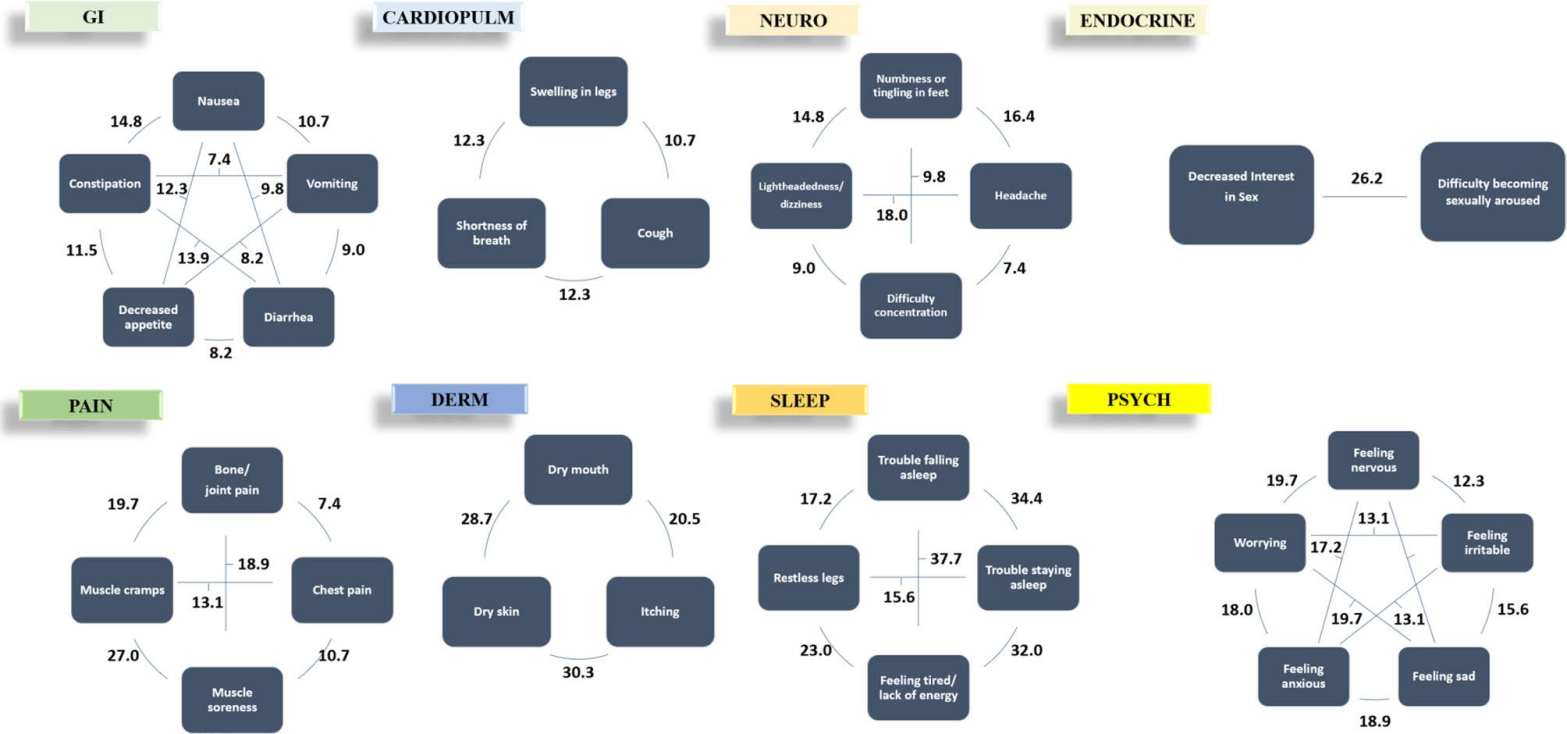
Prevalence of individual symptom pairings within symptom clusters in the overall cohort

We examined clusters of symptoms across seven categories, including the gastrointestinal, dermatologic, psychiatric, sleep, pain, neurologic, and endocrine systems (Fig. 3). The most prevalent pairings of symptoms within these clusters included (1) feeling tired and lack of energy + trouble falling asleep (37.7%), (2) trouble falling asleep + trouble staying asleep (34.4%), (3) feeling tired and lack of energy + trouble staying asleep (32.0%), (4) dry skin + itching (30.3%), and (5) dry skin + dry mouth (28.7%).

We also examined correlations of individual symptoms from the Dialysis Symptom Index. Using adjusted Pearson correlation, symptoms with the strongest correlations were (1) trouble falling asleep + trouble staying asleep (r = 0.86),( 2) decreased interest in sex + difficulty become sexually aroused (r = 0.74), (3) nausea + vomiting (r = 0.69), (4) feeling nervous + feeling sad (r = 0.63), and (5) feeling sad + feeling anxious (r = 0.61) (Supplementary Table 3). Similarly, using adjusted Spearman correlations, symptom pairings with the strongest correlations were (1) trouble falling asleep + trouble staying asleep (r = 0.81), (2) decreased interest in sex + difficulty become sexually aroused (r = 0.79), (3) nausea + vomiting (r = 0.59), (4) feeling sad + feeling anxious (r = 0.58), and (5) feeling nervous + feeling sad (r = 0.55) (Supplementary Table 4).

### Correlations between symptoms and key clinical characteristics

In a subcohort of 53 patients who had measurement of key laboratory parameters within six months prior to or within one month after their Dialysis Symptom Index survey assessment, we examined correlations between laboratory values with overall symptom severity and individual symptom severity scores with key laboratory parameters (Supplementary Table 5). We found that there were significant inverse correlations between lower hemoglobin levels with higher (worse) individual symptom scores for (1) feeling tired and lack of energy, (2) difficulty concentrating, and (3) dry skin. We also observed that lower transferrin saturation levels had significant inverse correlations with higher individual symptom scores for (1) muscle cramps, (2) restless legs or difficulty keeping legs still, and (3) muscle soreness, as well as higher overall symptom severity scores. We found that lower levels of dialysis adequacy ascertained by single-pool Kt/V and urea reduction ratio (URR) had significant inverse correlations with higher scores for (1) shortness of breath; we additionally found that lower URR levels were significantly correlated with higher scores for (2) vomiting, and (3) diarrhea, and (4) cough. Both lower serum albumin and higher phosphorus levels were significantly correlated with higher scores for diarrhea.

## Discussion/conclusion

In a well-characterized, multi-center prospective cohort of hemodialysis patients of diverse racial/ethnic background who underwent protocolized Dialysis Symptom Index surveys, we found that the overall symptom severity scores and prevalence of individual symptoms differed across race/ethnicity. Upon examining the most common unpleasant symptoms in the overall cohort and across racial/ethnic groups, the most frequently reported symptoms were those related to fatigue/energy, dermatologic conditions (i.e., dry skin, itching), and impaired sleep. Similarly, we found that the most prevalent clusters of symptoms occurred within these domains.

To date, a growing number of studies have utilized the Dialysis Symptom Index to quantify and characterize symptom burden in ESRD patients [6, 11–19]. In a seminal study that described the initial application of the Dialysis Symptom Index in US hemodialysis patients across three dialysis clinics, participants reported a median (IQR) number of symptoms of 9 (6, 13) and a median (IQR) overall symptom severity score of 25 (14, 42) [12]. Among the queried symptoms, over 50% of patients had dry skin, feelings of being tired or having lack of energy, itching, and/or bone/joint pain, with the highest mean severity scores for individual symptoms reported for bone/joint pain, chest pain, vomiting, difficulty becoming sexually aroused, and muscle cramps.

Subsequently, multiple international groups have sought to translate and/or cross-culturally adapt the Dialysis Symptom Index to their specific countries [6, 13, 15–17]. For example, in a study of 512 dialysis patients across 16 centers in the Netherlands, on average patients reported experiencing a mean ± SD of 11 ± 6 symptoms with an overall symptom severity score of 31 ± 22 based on a Dutch-version of the Dialysis Symptom Index [17]. In another study of 230 hemodialysis patients who underwent a Korean-version of the Dialysis Symptom Index across three hospitals in Seoul, nearly all participants (97.4%) reported having one or more symptoms, with the most prevalent physical symptoms including fatigue or weakness, dry skin, or weakness, and the most prevalent emotional symptoms including worrying, nervousness, and anxiety [16].

Despite these global efforts to advance our understanding of the scope of symptom burden in dialysis patients, there has been limited examination of how the prevalence, severity, and types of symptoms may vary among dialysis patients across diverse racial and ethnic backgrounds in the US. In a study of 78 Black and 82 White dialysis patients who underwent assessments of the Dialysis Symptom Index, Beck Depression Inventory, and Cognitive Depression Index, symptom burden was both considerable and comparable in both groups (median number of symptoms ~ 9 among both Black and White dialysis patients), and there were no observed racial differences in the overall severity of symptoms (median overall symptom severity score 25 and 24 in Black and White dialysis patients, respectively) [19]. However, in an exploratory analysis examining the importance attached to patients’ spiritual/religious beliefs, Black patients were more likely to report religious/spiritual beliefs as very important as compared with White patients (74% vs. 52%, respectively). Although importance of attachment to spiritual/religious beliefs was not correlated with symptom burden nor severity, in analyses stratified by race, correlations of the importance of spiritual/religious beliefs with Beck Depression Index and Cognitive Depression Index scores were significantly stronger among Black vs. White patients. In another study examining the interplay between cultural background and symptom burden among 75 dialysis patients from the US vs. 61 dialysis patients from Italy, Italian patients were considerably more likely to report physical and emotional symptoms than US patients (i.e., median [IQR] number of symptoms 14 [11, 16] vs. 9 [5, 12] in Italian vs. US patients, respectively) [18]. While these findings suggest a potential relationship between cultural background and adaptation to dialysis therapy, there is a paucity of data exploring differences in symptoms across ethnicity (i.e., Hispanic vs. Non-Hispanic patients) and other prevalent racial/ethnic groups in the US (i.e., Asian/Pacific Islanders).

To address this knowledge gap, we sought to characterize burden of unpleasant symptoms using the Dialysis Symptom Index in a diverse, multi-center US prospective hemodialysis cohort with representation of non-Hispanic White, White, Black, and Asian/Pacific Islander patients. We found that overall symptom severity scores and total number of reported symptoms tended to be higher among patients of non-Hispanic White and Hispanic race/ethnicity (i.e., suggesting higher self-reported symptom burden), whereas scores and symptom number tended to be lower in patients of Black and Asian/Pacific Islander race/ethnicity (i.e., suggesting lower self-reported symptom burden). With respect to types of symptoms, there were commonalities across racial/ethnic groups, with non-Hispanic White, Black, Hispanic White, Asian/Pacific Islander patients indicating that feeling tired or lack of energy was one of the most prevalent symptoms (i.e., 57–80% of patients in each racial/ethnic group). Additionally, Black, Hispanic, and Asian/Pacific Islander patients reported trouble falling asleep (i.e., 37.0–60.0% of patients in each racial/ethnic group) and dry mouth/dry skin (i.e., 40.7–100.0% of patients in each racial/ethnic group) among the most common symptoms. There were also some notable differences in the most prevalent symptoms reported across race/ethnicity. Whereas symptoms related to muscle pain (i.e., cramps, soreness) were more dominant in non-Hispanic White and Hispanic White patients (i.e., 43.4–80.0% of patients), symptoms related to sexual dysfunction (i.e., decreased interest in sex) were more frequently reported in Black and Asian/Pacific Islander patients (i.e., 40.7–57.1% of patients). Additionally, whereas symptoms related to cognition (i.e., difficulty concentrating) and psychologic status (i.e., worrying) were among the most common in non-Hispanic White and Hispanic White patients, respectively), these symptoms were less frequently reported in those of Black and Asian/Pacific Islander background. These distinctions highlight the need for further research exploring how underlying biologic, genetic, socioeconomic, psychologic, cultural, and spiritual/religious factors influence symptom burden across racial/ethnic groups [25–27], while also underscoring the importance of using a personalized approach in the symptom management of dialysis patients [28].

Another noteworthy finding of our study was the pervasiveness of certain symptoms across clusters. In studies of non-CKD patients (i.e., oncology), there has been growing recognition that symptom clusters, in which two or more concurrent symptoms may be inter-related with respect to their underlying etiology and/or potential to alter other symptoms, may have a more significant effect than isolated symptoms [1, 20]. While many dialysis patients experience multiple concurrent symptoms, there has been limited study of symptom clusters using comprehensive, validated symptom assessment surveys such as the Dialysis Symptom Index in this population. In this study, we found that the most prevalent pairings of symptoms within end-organ clusters tended to be those related to fatigue/energy, dermatologic conditions, and impaired sleep. Given the high prevalence of these unpleasant symptoms and their dominance within clusters, future research identifying therapies that can effectively target these symptoms may have a substantial effect on improving the health and well-being of a large proportion of dialysis patients.

While uremic toxins have traditionally been considered the main cause of CKD-associated symptoms, emerging data has shown that treatment of uremia by dialysis often fails to resolve them and can engender additional symptoms [5]. Notably, in sensitivity analyses examining the relationship between key laboratory parameters and symptoms, we found that lower levels of dialysis adequacy defined by spKt/V and URR correlated with greater severity of uremic (e.g., nausea) and volume overload (e.g., shortness of breath, cough) symptoms. Yet we also observed that worse anemia parameters defined by hemoglobin level and iron stores correlated with a broader range of symptoms, including those related to decreased energy (e.g., feeling tired or lack of energy), restless legs and muscle discomfort (e.g., restless legs or difficulty keeping legs still, muscle cramps, muscle soreness), impaired cognition (e.g., difficulty concentrating), and overall symptom burden (e.g., overall symptom severity score). As unpleasant symptoms and symptom clusters may ensue from a multitude of CKD-related complications, further studies are needed to precisely define their mechanistic underpinnings in the dialysis population.

The strengths of our study include its diverse cohort of hemodialysis patients who underwent protocolized Dialysis Symptom Index assessments; rigorous use of a symptom assessment tool that has been validated in the dialysis population; and comprehensive availability of detailed patient-level data collected in the outpatient setting. However, several limitations of our study bear mention. First, it is possible that patients who agreed to participate in the *MADRAD* study may be healthier than those who were not recruited, and may have thus had lower (better) symptom scores compared to the broader hemodialysis population. Second, while we had the opportunity to examine a well-characterized prospective cohort, the limited sample size of our study population likely precluded the ability to detect significant interactions on the basis of race/ethnicity (i.e., much larger sample size needed to estimate an interaction than to estimate a main effect). Third, given that our study examined Dialysis Symptom Index surveys at a single-point-in-time (i.e., baseline surveys collected at study entry), further longitudinal studies are needed to determine whether individual symptoms and symptom clusters change over time. Fourth, given that our recruitment was restricted to 16 outpatient dialysis units in Southern California, our findings may not be generalizable to other geographic regions where patients’ case-mix characteristics and dialysis practice patterns may differ. Finally, due to data limitations we were not able to examine the relationship between certain dialysis treatment characteristics (i.e., hemodialysis membrane, intra-dialytic medications) and symptom burden.

In conclusion, we observed a substantial burden of unpleasant symptoms in a diverse, prospective hemodialysis cohort, although there were notable differences in the prevalence of symptoms, overall symptom severity, and symptom types across racial/ethnic groups. We also found that symptoms related to fatigue/energy, dermatologic conditions, and impaired sleep were pervasive across racial/ethnic groups and symptom clusters. These findings underscore the critical need to incorporate standardized symptom assessments in the routine care of patients with CKD. Furthermore, future studies are needed to determine how patients’ underlying biologic, genetic, socioeconomic, psychologic, cultural, and spiritual/religious factors impact individual symptoms and symptom clusters, in order to identify targeted approaches that can ameliorate the high symptom burden of ESRD patients.

## Supplementary Information

Below is the link to the electronic supplementary material.

Supplementary file1 (DOCX 130 kb)

## Data Availability

The data that support the findings of this study are not publicly available due to containing information that could compromise the privacy of research participants. Further inquiries can be directed to the corresponding author.
