# Supplementary material for: Dialysis symptom index burden and symptom clusters in a prospective cohort of dialysis patients
Source: J Nephrol. 2022 Apr 16;35(5):1427–36. doi: 10.1007/s40620-022-01313-0 (PMC9217843; doi:10.1007/s40620-022-01313-0)
Supplement: Supplementary file 1 — Supplementary file1 (DOCX 130 kb) [file 40620_2022_1313_MOESM1_ESM.docx]

**SUPPLEMENTARY MATERIAL**

**Supplementary Table 1. Distribution of Dialysis Symptom Index scores in the overall cohort and across race/ethnicity.**

|  | **DIALYSIS SYMPTOM INDEX SCORE** | | |
| --- | --- | --- | --- |
|  | **Mean ± SD** | **Median (IQR)** | **Minimum-Maximum** |
| **Overall** | 28 ± 21 | 24 (11, 36) | 0-100 |
| **Non-Hispanic White** | 32 ± 23 | 26 (16, 54) | 6-68 |
| **Hispanic White** | 29 ± 21 | 25 (12, 37) | 2-90 |
| **Black** | 20 ± 14 | 22 (9, 30) | 0-45 |
| **Asian/Pacific Islander** | 26 ± 16 | 19 (14, 46) | 12-51 |

**Supplementary Table 2. Baseline characteristics across race/ethnicity.**

|  | **Overall** | **Non-Hispanic White** | **Hispanic White** | **Black** | **Asian/Pacific Islander** | **Other** | **p-value** |
| --- | --- | --- | --- | --- | --- | --- | --- |
| N (%) | 122 | 10 (8) | 76 (62) | 27 (22) | 7 (6) | 2 (2) | N/A |
| Age (mean±SD), years | 60±13 | 63±13 | 58±13 | 63±14 | 60±13 | 61±0 | 0.41 |
| Female (%) | 51 | 60 | 43 | 63 | 71 | 50 | 0.27 |
| Diabetes (%) | 67 | 50 | 76 | 44 | 86 | 50 | 0.01 |
| Vintage (mean±SD), months | 81±80 | 45±21 | 79±56 | 108±135 | 49±26 | 45±41 | 0.08 |
| Access (%)  AVG/AVF  Catheter | 93  7 | 90  10 | 93  7 | 96  4 | 86  14 | 100  0 | 0.55 |
| Marital (%)  Married  Single  Other | 37  37  26 | 0  60  40 | 47  26  26 | 22  59  19 | 29  29  43 | 50  50  0 | 0.005 |
| Insurance (%)  Medicare/MediCal  Kaiser, Private  Other | 84  11  5 | 90  10  0 | 86  11  4 | 74  19  7 | 86  0  14 | 100  0  0 | 0.66 |
| BMI (mean±SD), kg/m^2^ | 30.1±7.1 | 28.2±6.7 | 30.0±6.9 | 31.5±7.9 | 26.2±2.6 | 24.6±. | 0.53 |
| Post weight (mean±SD), lbs | 181±49 | 177±51 | 176±47 | 197±53 | 149±29 | 157±. | 0.29 |

Abbrev.: SD - standard deviation; AVG/AVF - arteriovenous graft/arteriovenous fistula; BMI – body mass index; kg/m^2^ – kilogram/meter squared; lbs – pounds.

**Supplementary Table 3. Correlations between symptoms using Pearson correlation adjusted for age, sex, and race.** *Red font text = p-value <0.05.*

|  | **Q1** | **Q2** | **Q3** | **Q4** | **Q5** | **Q6** | **Q7** | **Q8** | **Q9** | **Q10** | **Q11** | **Q12** | **Q13** | **Q14** | **Q15** | **Q16** | **Q17** | **Q18** | **Q19** | **Q20** | **Q21** | **Q22** | **Q23** | **Q24** | **Q25** | **Q26** | **Q27** | **Q28** | **Q29** | **Q30** |
| --- | --- | --- | --- | --- | --- | --- | --- | --- | --- | --- | --- | --- | --- | --- | --- | --- | --- | --- | --- | --- | --- | --- | --- | --- | --- | --- | --- | --- | --- | --- |
| **Q1. Constipation (R)** | **1.00** | **0.32** | **0.21** | **0.06** | **0.31** | **0.08** | **0.10** | **0.19** | **0.01** | **0.20** | **0.21** | **0.20** | **0.19** | **0.04** | **-0.09** | **0.10** | **0.01** | **-0.04** | **0.06** | **0.13** | **0.18** | **0.08** | **0.02** | **0.13** | **0.12** | **0.08** | **0.17** | **0.12** | **0.07** | **0.10** |
| **P-value** |  | <0.001 | 0.03 | 0.53 | <0.001 | 0.36 | 0.27 | 0.04 | 0.91 | 0.03 | 0.02 | 0.03 | 0.03 | 0.68 | 0.35 | 0.28 | 0.91 | 0.66 | 0.52 | 0.14 | 0.05 | 0.39 | 0.84 | 0.16 | 0.18 | 0.36 | 0.07 | 0.19 | 0.46 | 0.26 |
| **Q2. Nausea (R)** |  | **1.00** | **0.69** | **0.17** | **0.40** | **0.17** | **0.16** | **0.27** | **0.33** | **0.34** | **0.37** | **0.37** | **0.16** | **0.25** | **0.04** | **0.25** | **0.20** | **0.23** | **0.32** | **0.15** | **0.34** | **0.23** | **0.27** | **0.18** | **0.28** | **0.31** | **0.36** | **0.33** | **0.19** | **0.10** |
| **P-value** |  |  | <0.001 | 0.06 | <0.001 | 0.06 | 0.08 | 0.003 | <0.001 | <0.001 | <0.001 | <0.001 | 0.09 | 0.005 | 0.65 | 0.007 | 0.03 | 0.01 | <0.001 | 0.11 | <0.001 | 0.01 | 0.004 | 0.04 | 0.002 | <0.001 | <0.001 | <0.001 | 0.04 | 0.26 |
| **Q3. Vomiting (R)** |  |  | **1.00** | **0.27** | **0.39** | **0.22** | **0.06** | **0.34** | **0.22** | **0.34** | **0.24** | **0.25** | **0.21** | **0.19** | **-0.02** | **0.36** | **0.17** | **0.22** | **0.26** | **0.09** | **0.34** | **0.22** | **0.32** | **0.06** | **0.20** | **0.14** | **0.31** | **0.33** | **0.09** | **0.12** |
| **P-value** |  |  |  | 0.003 | <0.001 | 0.02 | 0.52 | <0.001 | 0.01 | <0.001 | 0.009 | 0.005 | 0.02 | 0.03 | 0.84 | <0.001 | 0.07 | 0.02 | 0.004 | 0.35 | <0.001 | 0.01 | <0.001 | 0.51 | 0.03 | 0.12 | <0.001 | <0.001 | 0.34 | 0.18 |
| **Q4. Diarrhea (R)** |  |  |  | **1.00** | **0.06** | **-0.02** | **0.27** | **0.44** | **0.29** | **0.11** | **0.05** | **0.29** | **0.05** | **0.22** | **-0.05** | **0.29** | **0.14** | **-0.006** | **0.25** | **0.14** | **0.22** | **0.14** | **0.21** | **0.29** | **0.27** | **0.31** | **0.19** | **0.21** | **0.02** | **0.08** |
| **P-value** |  |  |  |  | 0.54 | 0.87 | 0.003 | <0.001 | 0.002 | 0.22 | 0.60 | 0.001 | 0.56 | 0.01 | 0.57 | 0.001 | 0.13 | 0.95 | 0.006 | 0.14 | 0.02 | 0.13 | 0.02 | 0.001 | 0.004 | <0.001 | 0.04 | 0.02 | 0.81 | 0.39 |
| **Q5. Decreased appetite (R)** |  |  |  |  | **1.00** | **0.06** | **0.21** | **0.07** | **0.18** | **0.39** | **0.25** | **0.20** | **0.33** | **0.15** | **0.02** | **0.10** | **-0.06** | **0.14** | **0.20** | **0.14** | **0.14** | **0.19** | **0.15** | **0.14** | **0.15** | **0.05** | **0.26** | **0.19** | **0.16** | **0.20** |
| **P-value** |  |  |  |  |  | 0.53 | 0.02 | 0.43 | 0.06 | <0.001 | 0.007 | 0.03 | <0.001 | 0.11 | 0.84 | 0.29 | 0.54 | 0.14 | 0.03 | 0.15 | 0.13 | 0.04 | 0.10 | 0.14 | 0.11 | 0.61 | 0.005 | 0.04 | 0.08 | 0.03 |
| **Q6. Muscle Cramps (R)** |  |  |  |  |  | **1.00** | **0.17** | **0.07** | **0.12** | **0.25** | **0.16** | **0.20** | **0.12** | **0.10** | **0.21** | **0.18** | **0.42** | **0.46** | **0.10** | **0.08** | **0.15** | **0.02** | **0.04** | **0.07** | **0.05** | **-0.02** | **0.03** | **0.13** | **-0.07** | **-0.13** |
| **P-value** |  |  |  |  |  |  | 0.07 | 0.43 | 0.20 | 0.007 | 0.08 | 0.03 | 0.18 | 0.29 | 0.02 | 0.05 | <0.001 | <0.001 | 0.29 | 0.39 | 0.10 | 0.79 | 0.78 | 0.46 | 0.60 | 0.83 | 0.76 | 0.16 | 0.48 | 0.14 |
| **Q7. Swelling in Legs (R)** |  |  |  |  |  |  | **1.00** | **0.28** | **0.10** | **0.17** | **0.05** | **0.32** | **0.21** | **0.32** | **-0.0003** | **0.13** | **0.21** | **0.14** | **0.29** | **0.31** | **0.10** | **0.12** | **0.23** | **0.26** | **0.25** | **0.34** | **0.36** | **0.30** | **0.13** | **0.14** |
| **P-value** |  |  |  |  |  |  |  | 0.002 | 0.26 | 0.06 | 0.59 | <0.001 | 0.02 | <0.001 | >0.99 | 0.15 | 0.02 | 0.14 | 0.001 | <0.001 | 0.28 | 0.20 | 0.01 | 0.004 | 0.007 | <0.001 | <0.001 | 0.001 | 0.16 | 0.12 |
| **Q8. Shortness of breath (R)** |  |  |  |  |  |  |  | **1.00** | **0.21** | **0.13** | **0.08** | **0.31** | **0.19** | **0.06** | **-0.06** | **0.22** | **0.26** | **0.06** | **0.30** | **0.07** | **0.36** | **0.23** | **0.15** | **0.14** | **0.13** | **0.25** | **0.22** | **0.28** | **0.10** | **0.12** |
| **P-value** |  |  |  |  |  |  |  |  | 0.02 | 0.16 | 0.40 | <0.001 | 0.04 | 0.55 | 0.51 | 0.01 | 0.005 | 0.52 | 0.001 | 0.43 | <0.001 | 0.01 | 0.11 | 0.12 | 0.15 | 0.006 | 0.02 | 0.002 | 0.28 | 0.20 |
| **Q9. Lightheadedness or dizziness (R)** |  |  |  |  |  |  |  |  | **1.00** | **0.20** | **0.10** | **0.17** | **0.11** | **0.11** | **0.19** | **0.25** | **0.34** | **0.07** | **0.13** | **0.05** | **0.17** | **0.18** | **0.22** | **0.20** | **0.24** | **0.17** | **0.18** | **0.19** | **0.10** | **0.007** |
| **P-value** |  |  |  |  |  |  |  |  |  | 0.03 | 0.30 | 0.06 | 0.23 | 0.25 | 0.04 | 0.006 | <0.001 | 0.45 | 0.16 | 0.61 | 0.06 | 0.05 | 0.01 | 0.03 | 0.009 | 0.06 | 0.05 | 0.04 | 0.26 | 0.94 |
| **Q10. Restless legs or Difficulty keeping legs still (R)** |  |  |  |  |  |  |  |  |  | **1.00** | **0.34** | **0.30** | **0.42** | **0.28** | **0.05** | **0.11** | **0.12** | **0.32** | **0.16** | **0.21** | **0.17** | **0.28** | **0.31** | **0.32** | **0.30** | **-0.02** | **0.42** | **0.35** | **0.17** | **0.13** |
| **P-value** |  |  |  |  |  |  |  |  |  |  | <0.001 | <0.001 | <0.001 | 0.003 | 0.60 | 0.24 | 0.21 | <0.001 | 0.08 | 0.02 | 0.06 | 0.002 | <0.001 | <0.001 | <0.001 | 0.84 | <0.001 | <0.001 | 0.06 | 0.17 |
| **Q11. Numbness or tingling in feet (R)** |  |  |  |  |  |  |  |  |  |  | **1.00** | **0.12** | **0.08** | **0.24** | **0.22** | **-0.02** | **0.13** | **0.14** | **0.04** | **0.14** | **0.14** | **0.17** | **0.11** | **0.18** | **0.20** | **0.18** | **0.15** | **0.13** | **0.09** | **-0.01** |
| **P-value** |  |  |  |  |  |  |  |  |  |  |  | 0.20 | 0.41 | 0.008 | 0.02 | 0.87 | 0.15 | 0.12 | 0.69 | 0.13 | 0.14 | 0.07 | 0.25 | 0.04 | 0.03 | 0.05 | 0.10 | 0.16 | 0.31 | 0.89 |
| **Q12. Feeling tired or lack of energy (R)** |  |  |  |  |  |  |  |  |  |  |  | **1.00** | **0.29** | **0.52** | **0.29** | **0.29** | **0.17** | **0.34** | **0.45** | **0.30** | **0.22** | **0.32** | **0.32** | **0.35** | **0.36** | **0.33** | **0.49** | **0.44** | **0.17** | **0.19** |
| **P-value** |  |  |  |  |  |  |  |  |  |  |  |  | 0.002 | <0.001 | 0.001 | 0.002 | 0.06 | <0.001 | <0.001 | <0.001 | 0.01 | <0.001 | <0.001 | <0.001 | <0.001 | <0.001 | <0.001 | <0.001 | 0.06 | 0.04 |
| **Q13. Cough (R)** |  |  |  |  |  |  |  |  |  |  |  |  | **1.00** | **0.26** | **0.09** | **0.05** | **0.10** | **0.19** | **0.15** | **0.40** | **0.41** | **0.22** | **0.18** | **0.29** | **0.27** | **0.10** | **0.29** | **0.26** | **0.16** | **0.20** |
| **P-value** |  |  |  |  |  |  |  |  |  |  |  |  |  | 0.005 | 0.32 | 0.58 | 0.30 | 0.04 | 0.09 | <0.001 | <0.001 | 0.02 | 0.04 | 0.001 | 0.003 | 0.26 | 0.001 | 0.005 | 0.08 | 0.03 |
| **Q14. Dry mouth (R)** |  |  |  |  |  |  |  |  |  |  |  |  |  | **1.00** | **0.24** | **0.21** | **0.20** | **0.26** | **0.30** | **0.40** | **0.24** | **0.34** | **0.35** | **0.37** | **0.41** | **0.21** | **0.34** | **0.37** | **0.35** | **0.27** |
| **P-value** |  |  |  |  |  |  |  |  |  |  |  |  |  |  | 0.009 | 0.02 | 0.03 | 0.004 | <0.001 | <0.001 | 0.008 | <0.001 | <0.001 | <0.001 | <0.001 | 0.02 | <0.001 | <0.001 | <0.001 | 0.003 |
| **Q15. Bone or joint pain (R)** |  |  |  |  |  |  |  |  |  |  |  |  |  |  | **1.00** | **0.01** | **0.18** | **0.37** | **0.18** | **0.02** | **-0.02** | **0.25** | **0.15** | **0.17** | **0.17** | **0.03** | **0.21** | **0.13** | **0.10** | **0.001** |
| **P-value** |  |  |  |  |  |  |  |  |  |  |  |  |  |  |  | 0.91 | 0.05 | <0.001 | 0.06 | 0.82 | 0.84 | 0.007 | 0.10 | 0.07 | 0.06 | 0.76 | 0.02 | 0.14 | 0.30 | 0.99 |
| **Q16. Chest pain (R)** |  |  |  |  |  |  |  |  |  |  |  |  |  |  |  | **1.00** | **0.26** | **0.03** | **0.27** | **0.02** | **0.27** | **0.23** | **0.33** | **0.14** | **0.18** | **0.27** | **0.36** | **0.31** | **0.07** | **0.09** |
| **P-value** |  |  |  |  |  |  |  |  |  |  |  |  |  |  |  |  | 0.004 | 0.75 | 0.004 | 0.81 | 0.003 | 0.01 | <0.001 | 0.13 | 0.05 | 0.003 | <0.001 | <0.001 | 0.46 | 0.31 |
| **Q17. Headache (R)** |  |  |  |  |  |  |  |  |  |  |  |  |  |  |  |  | **1.00** | **0.22** | **0.004** | **0.10** | **0.11** | **0.07** | **0.16** | **0.14** | **0.14** | **0.10** | **0.06** | **0.14** | **0.20** | **-0.05** |
| **P-value** |  |  |  |  |  |  |  |  |  |  |  |  |  |  |  |  |  | 0.01 | 0.96 | 0.30 | 0.22 | 0.43 | 0.08 | 0.12 | 0.13 | 0.26 | 0.49 | 0.13 | 0.03 | 0.58 |
| **Q18. Muscle soreness (R)** |  |  |  |  |  |  |  |  |  |  |  |  |  |  |  |  |  | **1.00** | **0.21** | **0.27** | **0.14** | **0.19** | **0.13** | **0.10** | **0.11** | **0.06** | **0.16** | **0.16** | **0.16** | **0.13** |
| **P-value** |  |  |  |  |  |  |  |  |  |  |  |  |  |  |  |  |  |  | 0.02 | 0.003 | 0.14 | 0.04 | 0.16 | 0.28 | 0.22 | 0.51 | 0.09 | 0.09 | 0.08 | 0.17 |
| **Q19. Difficulty concentrating (R)** |  |  |  |  |  |  |  |  |  |  |  |  |  |  |  |  |  |  | **1.00** | **0.19** | **0.28** | **0.40** | **0.40** | **0.33** | **0.32** | **0.39** | **0.48** | **0.39** | **0.17** | **0.12** |
| **P-value** |  |  |  |  |  |  |  |  |  |  |  |  |  |  |  |  |  |  |  | 0.04 | 0.002 | <0.001 | <0.001 | <0.001 | <0.001 | <0.001 | <0.001 | <0.001 | 0.07 | 0.20 |
| **Q20. Dry skin (R)** |  |  |  |  |  |  |  |  |  |  |  |  |  |  |  |  |  |  |  | **1.00** | **0.35** | **0.21** | **0.05** | **0.22** | **0.17** | **0.20** | **0.12** | **0.20** | **0.20** | **0.13** |
| **P-value** |  |  |  |  |  |  |  |  |  |  |  |  |  |  |  |  |  |  |  |  | <0.001 | 0.02 | 0.58 | 0.02 | 0.07 | 0.03 | 0.19 | 0.03 | 0.03 | 0.15 |
| **Q21. Itching (R)** |  |  |  |  |  |  |  |  |  |  |  |  |  |  |  |  |  |  |  |  | **1.00** | **0.35** | **0.15** | **0.21** | **0.24** | **0.32** | **0.22** | **0.28** | **0.06** | **0.09** |
| **P-value** |  |  |  |  |  |  |  |  |  |  |  |  |  |  |  |  |  |  |  |  |  | <0.001 | 0.11 | 0.02 | 0.01 | <0.001 | 0.02 | 0.002 | 0.55 | 0.33 |
| **Q22. Worrying (R)** |  |  |  |  |  |  |  |  |  |  |  |  |  |  |  |  |  |  |  |  |  | **1.00** | **0.50** | **0.29** | **0.30** | **0.14** | **0.48** | **0.45** | **0.20** | **0.26** |
| **P-value** |  |  |  |  |  |  |  |  |  |  |  |  |  |  |  |  |  |  |  |  |  |  | <0.001 | 0.001 | 0.001 | 0.12 | <0.001 | <0.001 | 0.03 | 0.004 |
| **Q23. Feeling nervous (R)** |  |  |  |  |  |  |  |  |  |  |  |  |  |  |  |  |  |  |  |  |  |  | **1.00** | **0.35** | **0.35** | **0.28** | **0.63** | **0.60** | **0.22** | **0.23** |
| **P-value** |  |  |  |  |  |  |  |  |  |  |  |  |  |  |  |  |  |  |  |  |  |  |  | <0.001 | <0.001 | 0.002 | <0.001 | <0.001 | 0.02 | 0.01 |
| **Q24. Trouble falling asleep (R)** |  |  |  |  |  |  |  |  |  |  |  |  |  |  |  |  |  |  |  |  |  |  |  | **1.00** | **0.86** | **0.42** | **0.48** | **0.38** | **0.28** | **0.18** |
| **P-value** |  |  |  |  |  |  |  |  |  |  |  |  |  |  |  |  |  |  |  |  |  |  |  |  | <0.001 | <0.001 | <0.001 | <0.001 | 0.002 | 0.05 |
| **Q25. Trouble staying asleep (R)** |  |  |  |  |  |  |  |  |  |  |  |  |  |  |  |  |  |  |  |  |  |  |  |  | **1.00** | **0.48** | **0.54** | **0.40** | **0.35** | **0.25** |
| **P-value** |  |  |  |  |  |  |  |  |  |  |  |  |  |  |  |  |  |  |  |  |  |  |  |  |  | <0.001 | <0.001 | <0.001 | <0.001 | 0.006 |
| **Q26. Feeling irritable (R)** |  |  |  |  |  |  |  |  |  |  |  |  |  |  |  |  |  |  |  |  |  |  |  |  |  | **1.00** | **0.47** | **0.37** | **0.12** | **0.05** |
| **P-value** |  |  |  |  |  |  |  |  |  |  |  |  |  |  |  |  |  |  |  |  |  |  |  |  |  |  | <0.001 | <0.001 | 0.20 | 0.61 |
| **Q27. Feeling sad (R)** |  |  |  |  |  |  |  |  |  |  |  |  |  |  |  |  |  |  |  |  |  |  |  |  |  |  | **1.00** | **0.61** | **0.24** | **0.27** |
| **P-value** |  |  |  |  |  |  |  |  |  |  |  |  |  |  |  |  |  |  |  |  |  |  |  |  |  |  |  | <0.001 | 0.007 | 0.003 |
| **Q28. Feeling anxious (R)** |  |  |  |  |  |  |  |  |  |  |  |  |  |  |  |  |  |  |  |  |  |  |  |  |  |  |  | **1.00** | **0.26** | **0.26** |
| **P-value** |  |  |  |  |  |  |  |  |  |  |  |  |  |  |  |  |  |  |  |  |  |  |  |  |  |  |  |  | 0.004 | 0.004 |
| **Q29. Decreased interest in sex (R)** |  |  |  |  |  |  |  |  |  |  |  |  |  |  |  |  |  |  |  |  |  |  |  |  |  |  |  |  | **1.00** | **0.74** |
| **P-value** |  |  |  |  |  |  |  |  |  |  |  |  |  |  |  |  |  |  |  |  |  |  |  |  |  |  |  |  |  | <0.001 |
| **Q30. Difficulty becoming sexually aroused (R)** |  |  |  |  |  |  |  |  |  |  |  |  |  |  |  |  |  |  |  |  |  |  |  |  |  |  |  |  |  | **1.00** |
| **P-value** |  |  |  |  |  |  |  |  |  |  |  |  |  |  |  |  |  |  |  |  |  |  |  |  |  |  |  |  |  |  |

**Supplementary Table 4. Correlations between symptoms using Spearman correlation adjusted for age, sex, and race.** *Red font text = p-value <0.05.*

|  | **Q1** | **Q2** | **Q3** | **Q4** | **Q5** | **Q6** | **Q7** | **Q8** | **Q9** | **Q10** | **Q11** | **Q12** | **Q13** | **Q14** | **Q15** | **Q16** | **Q17** | **Q18** | **Q19** | **Q20** | **Q21** | **Q22** | **Q23** | **Q24** | **Q25** | **Q26** | **Q27** | **Q28** | **Q29** | **Q30** |
| --- | --- | --- | --- | --- | --- | --- | --- | --- | --- | --- | --- | --- | --- | --- | --- | --- | --- | --- | --- | --- | --- | --- | --- | --- | --- | --- | --- | --- | --- | --- |
| **Q1. Constipation (R)** | **1.00** | **0.35** | **0.17** | **0.15** | **0.24** | **0.09** | **0.15** | **0.22** | **0.10** | **0.23** | **0.15** | **0.14** | **0.19** | **-0.009** | **-0.06** | **0.17** | **0.05** | **-0.03** | **0.07** | **0.10** | **0.20** | **0.07** | **0.06** | **0.14** | **0.14** | **0.10** | **0.17** | **0.15** | **-0.02** | **0.02** |
| **P-value** |  | <0.001 | 0.06 | 0.10 | 0.01 | 0.34 | 0.11 | 0.02 | 0.27 | 0.01 | 0.09 | 0.12 | 0.04 | 0.93 | 0.49 | 0.07 | 0.57 | 0.78 | 0.43 | 0.29 | 0.03 | 0.46 | 0.53 | 0.12 | 0.12 | 0.30 | 0.06 | 0.11 | 0.85 | 0.80 |
| **Q2. Nausea (R)** |  | **1.00** | **0.59** | **0.22** | **0.41** | **0.15** | **0.20** | **0.32** | **0.35** | **0.35** | **0.33** | **0.36** | **0.25** | **0.19** | **0.09** | **0.28** | **0.22** | **0.22** | **0.26** | **0.13** | **0.34** | **0.22** | **0.25** | **0.18** | **0.29** | **0.33** | **0.37** | **0.28** | **0.10** | **0.06** |
| **P-value** |  |  | <0.001 | 0.02 | <0.001 | 0.10 | 0.03 | <0.001 | <0.001 | <0.001 | <0.001 | <0.001 | 0.005 | 0.04 | 0.34 | 0.002 | 0.02 | 0.01 | 0.005 | 0.17 | <0.001 | 0.02 | 0.007 | 0.05 | 0.001 | <0.001 | <0.001 | 0.002 | 0.26 | 0.53 |
| **Q3. Vomiting (R)** |  |  | **1.00** | **0.34** | **0.36** | **0.18** | **0.03** | **0.40** | **0.28** | **0.32** | **0.22** | **0.23** | **0.32** | **0.17** | **0.06** | **0.39** | **0.19** | **0.23** | **0.22** | **0.07** | **0.36** | **0.20** | **0.30** | **0.05** | **0.21** | **0.15** | **0.26** | **0.24** | **0.10** | **0.14** |
| **P-value** |  |  |  | <0.001 | <0.001 | 0.05 | 0.74 | <0.001 | 0.002 | <0.001 | 0.02 | 0.01 | <0.001 | 0.06 | 0.52 | <0.001 | 0.04 | 0.01 | 0.01 | 0.43 | <0.001 | 0.03 | <0.001 | 0.60 | 0.02 | 0.09 | 0.004 | 0.009 | 0.27 | 0.14 |
| **Q4. Diarrhea (R)** |  |  |  | **1.00** | **0.08** | **0.03** | **0.22** | **0.43** | **0.26** | **0.14** | **0.12** | **0.22** | **0.08** | **0.18** | **-0.07** | **0.30** | **0.18** | **-0.001** | **0.15** | **0.08** | **0.20** | **0.12** | **0.21** | **0.23** | **0.23** | **0.33** | **0.22** | **0.24** | **-0.01** | **0.09** |
| **P-value** |  |  |  |  | 0.41 | 0.73 | 0.01 | <0.001 | 0.005 | 0.14 | 0.19 | 0.02 | 0.39 | 0.05 | 0.48 | <0.001 | 0.05 | 0.99 | 0.11 | 0.39 | 0.03 | 0.20 | 0.02 | 0.01 | 0.01 | <0.001 | 0.02 | 0.008 | 0.95 | 0.34 |
| **Q5. Decreased appetite (R)** |  |  |  |  | **1.00** | **0.10** | **0.26** | **0.11** | **0.24** | **0.40** | **0.25** | **0.25** | **0.38** | **0.18** | **0.08** | **0.18** | **-0.02** | **0.20** | **0.19** | **0.21** | **0.18** | **0.22** | **0.15** | **0.12** | **0.14** | **0.11** | **0.24** | **0.17** | **0.17** | **0.22** |
| **P-value** |  |  |  |  |  | 0.30 | 0.004 | 0.22 | 0.009 | <0.001 | 0.006 | 0.007 | <0.001 | 0.05 | 0.42 | 0.05 | 0.83 | 0.03 | 0.04 | 0.02 | 0.04 | 0.02 | 0.11 | 0.18 | 0.14 | 0.24 | 0.007 | 0.06 | 0.07 | 0.01 |
| **Q6. Muscle Cramps (R)** |  |  |  |  |  | **1.00** | **0.14** | **0.09** | **0.13** | **0.24** | **0.14** | **0.19** | **0.15** | **0.07** | **0.23** | **0.24** | **0.38** | **0.46** | **0.10** | **0.09** | **0.15** | **0.04** | **0.02** | **0.08** | **0.04** | **0.01** | **0.05** | **0.13** | **-0.01** | **-0.08** |
| **P-value** |  |  |  |  |  |  | 0.12 | 0.34 | 0.15 | 0.008 | 0.12 | 0.04 | 0.09 | 0.46 | 0.01 | 0.008 | <0.001 | <0.001 | 0.26 | 0.36 | 0.10 | 0.66 | 0.81 | 0.38 | 0.68 | 0.89 | 0.61 | 0.15 | 0.95 | 0.37 |
| **Q7. Swelling in Legs (R)** |  |  |  |  |  |  | **1.00** | **0.26** | **0.10** | **0.17** | **0.04** | **0.33** | **0.20** | **0.31** | **0.03** | **0.20** | **0.15** | **0.17** | **0.32** | **0.29** | **0.10** | **0.15** | **0.24** | **0.26** | **0.24** | **0.39** | **0.35** | **0.24** | **0.15** | **0.17** |
| **P-value** |  |  |  |  |  |  |  | 0.004 | 0.26 | 0.07 | 0.67 | <0.001 | 0.03 | <0.001 | 0.78 | 0.03 | 0.09 | 0.06 | <0.001 | 0.001 | 0.29 | 0.10 | 0.009 | 0.004 | 0.01 | <0.001 | <0.001 | 0.008 | 0.10 | 0.06 |
| **Q8. Shortness of breath (R)** |  |  |  |  |  |  |  | **1.00** | **0.25** | **0.15** | **0.12** | **0.29** | **0.24** | **0.06** | **-0.03** | **0.27** | **0.24** | **0.09** | **0.25** | **0.09** | **0.40** | **0.19** | **0.18** | **0.13** | **0.13** | **0.31** | **0.20** | **0.30** | **0.07** | **0.10** |
| **P-value** |  |  |  |  |  |  |  |  | 0.007 | 0.11 | 0.19 | 0.001 | 0.008 | 0.49 | 0.74 | 0.003 | 0.008 | 0.35 | 0.007 | 0.34 | <0.001 | 0.04 | 0.06 | 0.14 | 0.17 | <0.001 | 0.03 | <0.001 | 0.44 | 0.30 |
| **Q9. Lightheadedness or dizziness (R)** |  |  |  |  |  |  |  |  | **1.00** | **0.19** | **0.09** | **0.20** | **0.18** | **0.12** | **0.23** | **0.28** | **0.39** | **0.08** | **0.12** | **0.04** | **0.16** | **0.15** | **0.22** | **0.23** | **0.25** | **0.22** | **0.19** | **0.20** | **0.06** | **0.02** |
| **P-value** |  |  |  |  |  |  |  |  |  | 0.04 | 0.3064 | 0.03 | 0.05 | 0.20 | 0.01 | 0.002 | <0.001 | 0.38 | 0.18 | 0.70 | 0.09 | 0.10 | 0.01 | 0.01 | 0.005 | 0.02 | 0.04 | 0.03 | 0.53 | 0.84 |
| **Q10. Restless legs or Difficulty keeping legs still (R)** |  |  |  |  |  |  |  |  |  | **1.00** | **0.3139** | **0.30** | **0.36** | **0.26** | **0.09** | **0.19** | **0.13** | **0.30** | **0.18** | **0.17** | **0.13** | **0.23** | **0.25** | **0.29** | **0.27** | **0.01** | **0.38** | **0.29** | **0.15** | **0.08** |
| **P-value** |  |  |  |  |  |  |  |  |  |  | <0.001 | 0.001 | <0.001 | 0.005 | 0.35 | 0.04 | 0.15 | 0.001 | 0.05 | 0.06 | 0.15 | 0.01 | 0.007 | 0.001 | 0.003 | 0.92 | <0.001 | 0.002 | 0.10 | 0.39 |
| **Q11. Numbness or tingling in feet (R)** |  |  |  |  |  |  |  |  |  |  | **1.00** | **0.10** | **0.10** | **0.22** | **0.21** | **0.02** | **0.15** | **0.12** | **0.01** | **0.12** | **0.14** | **0.15** | **0.09** | **0.14** | **0.17** | **0.18** | **0.09** | **0.10** | **0.07** | **-0.04** |
| **P-value** |  |  |  |  |  |  |  |  |  |  |  | 0.29 | 0.27 | 0.02 | 0.02 | 0.81 | 0.10 | 0.19 | 0.93 | 0.21 | 0.13 | 0.11 | 0.33 | 0.14 | 0.06 | 0.05 | 0.33 | 0.28 | 0.45 | 0.64 |
| **Q12. Feeling tired or lack of energy (R)** |  |  |  |  |  |  |  |  |  |  |  | **1.00** | **0.29** | **0.50** | **0.26** | **0.28** | **0.16** | **0.32** | **0.43** | **0.30** | **0.24** | **0.30** | **0.27** | **0.35** | **0.36** | **0.31** | **0.45** | **0.44** | **0.21** | **0.21** |
| **P-value** |  |  |  |  |  |  |  |  |  |  |  |  | 0.002 | <0.001 | 0.004 | 0.002 | 0.08 | <0.001 | <0.001 | 0.001 | 0.01 | 0.001 | 0.003 | <0.001 | <0.001 | <0.001 | <0.001 | <0.001 | 0.02 | 0.02 |
| **Q13. Cough (R)** |  |  |  |  |  |  |  |  |  |  |  |  | **1.00** | **0.17** | **0.10** | **0.10** | **0.15** | **0.18** | **0.24** | **0.36** | **0.41** | **0.22** | **0.19** | **0.1797** | **0.18** | **0.14** | **0.26** | **0.23** | **0.05** | **0.08** |
| **P-value** |  |  |  |  |  |  |  |  |  |  |  |  |  | 0.06 | 0.27 | 0.26 | 0.11 | 0.05 | 0.01 | <0.001 | <0.001 | 0.01 | 0.04 | 0.05 | 0.06 | 0.12 | 0.004 | 0.01 | 0.60 | 0.38 |
| **Q14. Dry mouth (R)** |  |  |  |  |  |  |  |  |  |  |  |  |  | **1.00** | **0.17** | **0.19** | **0.17** | **0.20** | **0.25** | **0.38** | **0.21** | **0.32** | **0.32** | **0.35** | **0.40** | **0.25** | **0.31** | **0.34** | **0.27** | **0.22** |
| **P-value** |  |  |  |  |  |  |  |  |  |  |  |  |  |  | 0.06 | 0.04 | 0.06 | 0.03 | 0.006 | <0.001 | 0.02 | <0.001 | <0.001 | <0.001 | <0.001 | 0.007 | <0.001 | <0.001 | 0.003 | 0.02 |
| **Q15. Bone or joint pain (R)** |  |  |  |  |  |  |  |  |  |  |  |  |  |  | **1.00** | **0.02** | **0.19** | **0.33** | **0.13** | **-0.001** | **-0.04** | **0.18** | **0.12** | **0.14** | **0.14** | **0.02** | **0.14** | **0.10** | **0.08** | **-0.03** |
| **P-value** |  |  |  |  |  |  |  |  |  |  |  |  |  |  |  | 0.83 | 0.04 | <0.001 | 0.15 | 0.99 | 0.67 | 0.06 | 0.19 | 0.13 | 0.14 | 0.84 | 0.14 | 0.27 | 0.37 | 0.74 |
| **Q16. Chest pain (R)** |  |  |  |  |  |  |  |  |  |  |  |  |  |  |  | **1.00** | **0.31** | **0.09** | **0.25** | **0.04** | **0.24** | **0.19** | **0.35** | **0.12** | **0.19** | **0.36** | **0.40** | **0.33** | **0.001** | **0.02** |
| **P-value** |  |  |  |  |  |  |  |  |  |  |  |  |  |  |  |  | <0.001 | 0.35 | 0.005 | 0.68 | 0.008 | 0.04 | <0.001 | 0.19 | 0.04 | <0.001 | <0.001 | <0.001 | >0.99 | 0.81 |
| **Q17. Headache (R)** |  |  |  |  |  |  |  |  |  |  |  |  |  |  |  |  | **1.00** | **0.15** | **0.004** | **0.10** | **0.12** | **0.05** | **0.21** | **0.15** | **0.16** | **0.19** | **0.05** | **0.15** | **0.05** | **-0.12** |
| **P-value** |  |  |  |  |  |  |  |  |  |  |  |  |  |  |  |  |  | 0.10 | 0.97 | 0.29 | 0.21 | 0.58 | 0.02 | 0.11 | 0.08 | 0.04 | 0.58 | 0.10 | 0.58 | 0.20 |
| **Q18. Muscle soreness (R)** |  |  |  |  |  |  |  |  |  |  |  |  |  |  |  |  |  | **1.00** | **0.24** | **0.25** | **0.15** | **0.19** | **0.12** | **0.12** | **0.14** | **0.07** | **0.15** | **0.16** | **0.17** | **0.13** |
| **P-value** |  |  |  |  |  |  |  |  |  |  |  |  |  |  |  |  |  |  | 0.008 | 0.006 | 0.10 | 0.04 | 0.19 | 0.18 | 0.14 | 0.48 | 0.11 | 0.09 | 0.06 | 0.17 |
| **Q19. Difficulty concentrating (R)** |  |  |  |  |  |  |  |  |  |  |  |  |  |  |  |  |  |  | **1.00** | **0.19** | **0.28** | **0.36** | **0.32** | **0.36** | **0.31** | **0.36** | **0.47** | **0.34** | **0.13** | **0.09** |
| **P-value** |  |  |  |  |  |  |  |  |  |  |  |  |  |  |  |  |  |  |  | 0.03 | 0.002 | <0.001 | <0.001 | <0.001 | <0.001 | <0.001 | <0.001 | <0.001 | 0.17 | 0.34 |
| **Q20. Dry skin (R)** |  |  |  |  |  |  |  |  |  |  |  |  |  |  |  |  |  |  |  | **1.00** | **0.29** | **0.20** | **0.02** | **0.19** | **0.13** | **0.22** | **0.13** | **0.17** | **0.20** | **0.19** |
| **P-value** |  |  |  |  |  |  |  |  |  |  |  |  |  |  |  |  |  |  |  |  | 0.001 | 0.03 | 0.83 | 0.04 | 0.15 | 0.02 | 0.17 | 0.06 | 0.03 | 0.04 |
| **Q21. Itching (R)** |  |  |  |  |  |  |  |  |  |  |  |  |  |  |  |  |  |  |  |  | **1.00** | **0.32** | **0.13** | **0.19** | **0.21** | **0.37** | **0.24** | **0.32** | **0.06** | **0.11** |
| **P-value** |  |  |  |  |  |  |  |  |  |  |  |  |  |  |  |  |  |  |  |  |  | <0.001 | 0.15 | 0.04 | 0.02 | <0.001 | 0.009 | <0.001 | 0.51 | 0.24 |
| **Q22. Worrying (R)** |  |  |  |  |  |  |  |  |  |  |  |  |  |  |  |  |  |  |  |  |  | **1.00** | **0.46** | **0.26** | **0.26** | **0.19** | **0.44** | **0.41** | **0.19** | **0.26** |
| **P-value** |  |  |  |  |  |  |  |  |  |  |  |  |  |  |  |  |  |  |  |  |  |  | <0.001 | 0.005 | 0.005 | 0.04 | <0.001 | <0.001 | 0.04 | 0.005 |
| **Q23. Feeling nervous (R)** |  |  |  |  |  |  |  |  |  |  |  |  |  |  |  |  |  |  |  |  |  |  | **1.00** | **0.29** | **0.31** | **0.30** | **0.55** | **0.53** | **0.14** | **0.13** |
| **P-value** |  |  |  |  |  |  |  |  |  |  |  |  |  |  |  |  |  |  |  |  |  |  |  | 0.002 | <0.001 | 0.001 | <0.001 | <0.001 | 0.12 | 0.17 |
| **Q24. Trouble falling asleep (R)** |  |  |  |  |  |  |  |  |  |  |  |  |  |  |  |  |  |  |  |  |  |  |  | **1.00** | **0.81** | **0.43** | **0.44** | **0.36** | **0.20** | **0.12** |
| **P-value** |  |  |  |  |  |  |  |  |  |  |  |  |  |  |  |  |  |  |  |  |  |  |  |  | <0.001 | <0.001 | <0.001 | <0.001 | 0.03 | 0.21 |
| **Q25. Trouble staying asleep (R)** |  |  |  |  |  |  |  |  |  |  |  |  |  |  |  |  |  |  |  |  |  |  |  |  | **1.0000** | **0.50** | **0.52** | **0.38** | **0.26** | **0.17** |
| **P-value** |  |  |  |  |  |  |  |  |  |  |  |  |  |  |  |  |  |  |  |  |  |  |  |  |  | <0.001 | <0.001 | <0.001 | 0.005 | 0.06 |
| **Q26. Feeling irritable (R)** |  |  |  |  |  |  |  |  |  |  |  |  |  |  |  |  |  |  |  |  |  |  |  |  |  | **1.00** | **0.47** | **0.37** | **0.08** | **0.01** |
| **P-value** |  |  |  |  |  |  |  |  |  |  |  |  |  |  |  |  |  |  |  |  |  |  |  |  |  |  | <0.001 | <0.001 | 0.39 | 0.91 |
| **Q27. Feeling sad (R)** |  |  |  |  |  |  |  |  |  |  |  |  |  |  |  |  |  |  |  |  |  |  |  |  |  |  | **1.00** | **0.58** | **0.10** | **0.09** |
| **P-value** |  |  |  |  |  |  |  |  |  |  |  |  |  |  |  |  |  |  |  |  |  |  |  |  |  |  |  | <0.001 | 0.30 | 0.33 |
| **Q28. Feeling anxious (R)** |  |  |  |  |  |  |  |  |  |  |  |  |  |  |  |  |  |  |  |  |  |  |  |  |  |  |  | **1.00** | **0.12** | **0.11** |
| **P-value** |  |  |  |  |  |  |  |  |  |  |  |  |  |  |  |  |  |  |  |  |  |  |  |  |  |  |  |  | 0.19 | 0.24 |
| **Q29. Decreased interest in sex (R)** |  |  |  |  |  |  |  |  |  |  |  |  |  |  |  |  |  |  |  |  |  |  |  |  |  |  |  |  | **1.00** | **0.79** |
| **P-value** |  |  |  |  |  |  |  |  |  |  |  |  |  |  |  |  |  |  |  |  |  |  |  |  |  |  |  |  |  | <0.001 |
| **Q30. Difficulty becoming sexually aroused (R)** |  |  |  |  |  |  |  |  |  |  |  |  |  |  |  |  |  |  |  |  |  |  |  |  |  |  |  |  |  | **1.00** |
| **P-value** |  |  |  |  |  |  |  |  |  |  |  |  |  |  |  |  |  |  |  |  |  |  |  |  |  |  |  |  |  |  |

**Supplementary Table 5. Correlations between overall symptom severity score and individual symptom severity with key laboratory parameters.*** *P-values shown in italics. Red font text = p-value <0.05.*

|  | **Hemoglobin**  **R (p-value)** | **Tsat**  **R (p-value)** | **spKt/V**  **R (p-value)** | **URR**  **R (p-value)** | **Albumin**  **R (p-value)** | **Phos**  **R (p-value)** | **Sodium**  **R (p-value)** | **Creatinine**  **R (p-value)** |
| --- | --- | --- | --- | --- | --- | --- | --- | --- |
| **Overall DSI score** | **-0.20**  *(p=0.15)* | **-0.29**  *(p=0.03)* | **-0.19**  *(p=0.18)* | **-0.25**  *(p=0.07)* | **-0.02**  *(p=0.88)* | **0.02**  *(p=0.91)* | **-0.09**  *(p=0.54)* | **-0.15**  *(p=0.30)* |
| **1. Constipation** | **0.14**  *(p=0.33)* | **-0.13**  *(p=0.36)* | **-0.10**  *(p=0.49)* | **-0.06**  *(p=0.70)* | **0.10**  *(p=0.48)* | **-0.06**  *(p=0.65)* | **0.02**  *(p=0.89)* | **-0.15**  *(p=0.29)* |
| **2. Nausea** | **-0.15**  *(p=0.28)* | **-0.27**  *(p=0.05)* | **-0.05**  *(p=0.72)* | **-0.12**  *(p=0.40)* | **0.02**  *(p=0.88)* | **0.15**  *(p=0.27)* | **-0.12**  *(p=0.41)* | **-0.03**  *(p=0.85)* |
| **3. Vomiting** | **-0.04**  *(p=0.76)* | **-0.25**  *(p=0.07)* | **-0.23**  *(p=0.10)* | **-0.31**  *(p=0.03)* | **-0.07**  *(p=0.63)* | **0.10**  *(p=0.46)* | **-0.02**  *(p=0.88)* | **-0.10**  *(p=0.49)* |
| **4. Diarrhea** | **-0.08**  *(p=0.57)* | **0.14**  *(p=0.32)* | **-0.25**  *(p=0.08)* | **-0.34**  *(p=0.01)* | **-0.37**  *(p=0.01)* | **0.33**  *(p=0.02)* | **-0.27**  *(p=0.06)* | **-0.08**  *(p=0.56)* |
| **5. Decreased appetite** | **-0.13**  *(p=0.36)* | **-0.26**  *(p=0.07)* | **-0.17**  *(p=0.22)* | **-0.24**  *(p=0.09)* | **0.16**  *(p=0.26)* | **-0.10**  *(p=0.48)* | **0.18**  *(p=0.20)* | **-0.03**  *(p=0.82)* |
| **6. Muscle Cramps** | **0.04**  *(p=0.80)* | **-0.29**  *(p=0.04)* | **-0.10**  *(p=0.48)* | **-0.14**  *(p=0.33)* | **0.24**  *(p=0.09)* | **0.07**  *(p=0.62)* | **-0.05**  *(p=0.75)* | **-0.16**  *(p=0.26)* |
| **7. Swelling in Legs** | **-0.19**  *(p=0.17)* | **-0.03**  *(p=0.81)* | **-0.15**  *(p=0.29)* | **-0.22**  *(p=0.12)* | **0.16**  *(p=0.24)* | **0.04**  *(p=0.76)* | **-0.03**  *(p=0.81)* | **-0.06**  *(p=0.65)* |
| **8. Shortness of breath** | **0.11**  *(p=0.42)* | **-0.10**  *(p=0.47)* | **-0.39**  *(p<0.001)* | **-0.43**  *(p<0.001)* | **-0.01**  *(p=0.96)* | **0.17**  *(p=0.24)* | **0.08**  *(p=0.57)* | **0.03**  *(p=0.86)* |
| **9. Lightheadedness or dizziness** | **-0.23**  *(p=0.09)* | **-0.14**  *(p=0.31)* | **-0.20**  *(p=0.16)* | **-0.26**  *(p=0.06)* | **0.10**  *(p=0.49)* | **0.21**  *(p=0.13)* | **-0.22**  *(p=0.12)* | **0.04**  *(p=0.76)* |
| **10.** **Restless legs or difficulty keeping legs still** | **0.01**  *(p=0.94)* | **-0.30**  *(p=0.03)* | **-0.004**  *(p=0.98)* | **-0.04**  *(p=0.75)* | **0.06**  (p=0.66) | **-0.04**  *(p=0.78)* | **-0.03**  *(p=0.84)* | **-0.19**  *(p=0.17)* |
| **11. Numbness or tingling in feet** | **-0.04**  *(p=0.80)* | **-0.14**  *(p=0.31)* | **-0.01**  *(p=0.92)* | **-0.05**  *(p=0.72)* | **-0.02**  *(p=0.88)* | **0.04**  *(p=0.79)* | **0.17**  *(p=0.23)* | **-0.10**  *(p=0.47)* |
| **12.** **Feeling tired or lack of energy** | **-0.31**  *(p=0.02)* | **-0.19**  *(p=0.17)* | **-0.04**  *(p=0.76)* | **-0.07**  *(p=0.63)* | **-0.20**  *(p=0.14)* | **-0.09**  *(p=0.55)* | **-0.23**  *(p=0.09)* | **-0.14**  *(p=0.31)* |
| **13. Cough** | **-0.08**  *(p=0.55)* | **-0.23**  *(p=0.11)* | **-0.26**  *(p=0.07)* | **-0.32**  *(p=0.02)* | **0.09**  *(p=0.53)* | **-0.09**  *(p=0.51)* | **0.17**  *(p=0.23)* | **0.23**  *(p=0.09)* |
| **14. Dry mouth** | **-0.26**  *(p=0.06)* | **-0.13**  *(p=0.37)* | **0.01**  *(p=0.97)* | **-0.05**  *(p=0.74)* | **-0.16**  *(p=0.26)* | **-0.08**  *(p=0.56)* | **-0.16**  *(p=0.25)* | **-0.14**  *(p=0.31)* |
| **15. Bone or joint pain** | **-0.18**  *(p=0.19)* | **0.05**  *(p=0.75)* | **-0.23**  *(p=0.10)* | **-0.24**  *(p=0.08)* | **-0.03**  *(p=0.83)* | **-0.01**  *(p=0.95)* | **-0.04**  *(p=0.76)* | **-0.10**  *(p=0.48)* |
| **16. Chest pain** | **-0.16**  *(p=0.25)* | **-0.07**  *(p=0.64)* | **0.18**  *(p=0.21)* | **0.18**  *(p=0.19)* | **0.09**  *(p=0.52)* | **0.03**  *(p=0.81)* | **-0.07**  *(p=0.61)* | **-0.18**  *(p=0.21)* |
| **17. Headache** | **0.08**  *(p=0.59)* | **-0.19**  *(p=0.18)* | **-0.20**  *(p=0.16)* | **-0.26**  *(p=0.06)* | **0.03**  *(p=0.85)* | **0.04**  *(p=0.77)* | **0.07**  *(p=0.62)* | **-0.004**  *(p=0.98)* |
| **18. Muscle soreness** | **0.14**  *(p=0.30)* | **-0.30**  *(p=0.03)* | **-0.14**  *(p=0.31)* | **-0.18**  *(p=0.19)* | **0.18**  *(p=0.19)* | **0.03**  *(p=0.84)* | **0.10**  *(p=0.48)* | **-0.04**  *(p=0.79)* |
| **19. Difficulty concentrating** | **-0.33**  *(p=0.02)* | **-0.19**  *(p=0.18)* | **-0.06**  *(p=0.65)* | **-0.08**  *(p=0.57)* | **0.02**  *(p=0.88)* | **-0.002**  *(p=0.99)* | **-0.04**  *(p=0.80)* | **0.01**  *(p=0.92)* |
| **20. Dry skin** | **-0.31**  *(p=0.02)* | **0.10**  *(p=0.48)* | **0.14**  *(p=0.33)* | **0.09**  *(p=0.53)* | **-0.17**  *(p=0.23)* | **-0.15**  *(p=0.27)* | **-0.27**  *(p=0.05)* | **-0.14**  *(p=0.31)* |
| **21. Itching** | **-0.04**  *(p=0.81)* | **-0.04**  *(p=0.75)* | **0.06**  *(p=0.67)* | **0.01**  *(p=0.94)* | **-0.23**  *(p=0.10)* | **0.11**  *(p=0.43)* | **-0.14**  *(p=0.31)* | **-0.03**  (p=0.86) |
| **22. Worrying** | **-0.04**  *(p=0.80)* | **-0.19**  *(p=0.17)* | **-0.10**  *(p=0.49)* | **-0.10**  *(p=0.46)* | **0.04**  *(p=0.77)* | **0.04**  *(p=0.76)* | **0.10**  *(p=0.50)* | **-0.12**  *(p=0.41)* |
| **23. Feeling nervous** | **-0.27**  *(p=0.05)* | **-0.18**  *(p=0.19)* | **-0.10**  *(p=0.51)* | **-0.12**  *(p=0.40)* | **0.02**  *(p=0.87)* | **-0.03**  *(p=0.85)* | **0.06**  *(p=0.65)* | **0.004**  *(p=0.98)* |
| **24. Trouble falling asleep** | **-0.24**  *(p=0.09)* | **-0.22**  *(p=0.12)* | **-0.14**  *(p=0.32)* | **-0.15**  *(p=0.28)* | **-0.11**  *(p=0.43)* | **-0.17**  *(p=0.22)* | **-0.16**  *(p=0.26)* | **-0.17**  *(p=0.23)* |
| **25. Trouble staying asleep** | **-0.26**  *(p=0.06)* | **-0.29**  *(p=0.04)* | **-0.17**  *(p=0.24)* | **-0.17**  *(p=0.22)* | **-0.21**  *(p=0.14)* | **-0.19**  *(p=0.18)* | **-0.23**  *(p=0.10)* | **-0.23**  *(p=0.10)* |
| **26. Feeling irritable** | **0.02**  *(p=0.87)* | **-0.18**  *(p=0.20)* | **-0.22**  *(p=0.11)* | **-0.26**  *(p=0.06)* | **0.01**  *(p=0.94)* | **0.11**  *(p=0.45)* | **0.05**  *(p=0.73)* | **0.13**  *(p=0.34)* |
| **27. Feeling sad** | **-0.11**  *(p=0.42)* | **-0.24**  *(p=0.09)* | **-0.08**  *(p=0.56)* | **-0.12**  *(p=0.39)* | **0.03**  *(p=0.82)* | **-0.03**  *(p=0.84)* | **-0.11**  *(p=0.42)* | **-0.08**  *(p=0.56)* |
| **28. Feeling anxious** | **-0.05**  *(p=0.75)* | **-0.20**  *(p=0.15)* | **-0.08**  *(p=0.58)* | **-0.09**  *(p=0.51)* | **0.01**  *(p=0.95)* | **-0.07**  *(p=0.60)* | **0.12**  *(p=0.40)* | **-0.10**  *(p=0.48)* |
| **29. Decreased interest in sex** | **-0.06**  *(p=0.68)* | **0.003**  *(p=0.98)* | **0.14**  *(p=0.31)* | **0.16**  *(p=0.25)* | **0.10**  *(p=0.49)* | **0.06**  *(p=0.69)* | **-0.02**  *(p=0.88)* | **-0.02**  *(p=0.91)* |
| **30. Difficulty becoming sexually aroused** | **0.02**  *(p=0.88)* | **-0.03**  *(p=0.82)* | **0.04**  *(p=0.80)* | **0.07**  *(p=0.61)* | **0.09**  *(p=0.53)* | **0.10**  *(p=0.49)* | **-0.16**  *(p=0.27)* | **-0.13**  *(p=0.35)* |

*Abbrev.: Tsat, transferrin saturation; spKt/V, single-pool Kt/V; URR, urea reduction ratio; albumin, serum albumin; phos, phosphorus; creatinine, serum creatinine.*
